# Telehealth experience in supplementary health: monitoring health care

**DOI:** 10.1590/0034-7167-2025-0032

**Published:** 2025-12-08

**Authors:** Lucas Cardoso dos Santos, Glaucia Cristina de Oliveira, Natália Tatiani Gonçalves Brito, Lislaine Aparecida Fracolli

**Affiliations:** I Universidade de São Paulo. São Paulo, São Paulo, Brazil; II Sociedade Beneficente Israelita Brazileira Albert Einstein. São Paulo, São Paulo, Brazil

**Keywords:** Telemedicine, Supplemental Health, Continuity of Patient Care, Medical Records Systems Computerized, Information Technology., Telemedicina, Salud Complementaria, Continuidad de la Atención al Paciente, Sistemas de Registros Médicos Computarizados, Tecnología de la Información.

## Abstract

**Objectives:**

to report the experience of telehealth in monitoring healthcare in the context of supplementary health.

**Methods:**

an experience report developed in telehealth service in supplementary health for the years 2022 to 2024.

**Results:**

Information and Communication Technologies have expanded the provision of care, access to health through telehealth, patients seeking care provided by nurses, and professionals’ job satisfaction. The use of care protocols and digital technologies has enabled the construction of indicators and dashboards for care management. Challenges encountered were connection problems, lack of skills with digital technologies, lack of training and literacy in digital health, and lack of understanding of the role of nurses by patients, other services, and professionals.

**Final Considerations:**

telehealth contributes to healthcare qualification, highlighting digital health as a powerful field of action for nurses.

## RESUMEN

**Objetivos:**

relatar la experiencia de la telesalud en el seguimiento de la atención a la salud en el contexto de la salud complementaria.

**Métodos:**

informe de experiencia desarrollado en servicio de telesalud en salud complementaria para los años 2022 a 2024.

**Resultados:**

las Tecnologías de la Información y la Comunicación ampliaron la prestación de cuidados, el acceso a la salud a través de la telesalud, la búsqueda de los pacientes por la atención brindada por enfermeros y la satisfacción laboral de los profesionales. El uso de protocolos de atención y tecnologías digitales permitió construir indicadores y tableros de control para la gestión del cuidado. Los desafíos encontrados fueron problemas de conexión, falta de habilidades con las tecnologías digitales, falta de capacitación y alfabetización en salud digital y falta de comprensión del papel de la enfermera por parte del paciente, otros servicios y profesionales.

**Consideraciones Finales:**

la telesalud contribuye a la cualificación de la atención en salud, destacando la salud digital como un poderoso campo de acción para las enfermeras.

## INTRODUCTION

The use of digital technologies began during the First World War, with the use of radio to mediate communication between physicians on the front lines and hospitals. Further advances followed in the 1960s, with the radio communication system to monitor astronauts’ vital signs, in the 1970s, with the use of online standards for banking transactions, and in the 1980s, with industrial processes and later in the health sector^(1)^.

In Brazil, Information and Communication Technologies (ICTs) have been used since 1970 with the implementation of the Mortality Information System. After the creation of the Brazilian Health System (In Portuguese, *Sistema Único de Saúde* - SUS) Department of Information Technology in 1991, other systems were implemented, adding to other more recent milestones for digital health such as the enactment of the Brazilian National Policy for Technological Innovation in Health (2017), the General Law for the Protection of Personal Data (2018), the Brazilian National Digital Health Network (2020), the Digital Health Strategy for Brazil (2020), the Brazilian National Information and Information Policy (2021) and the Department of Information and Digital Health (2023)^(2)^.

Digital health incorporates advances in technology, the Internet of Things, artificial intelligence and Big Data^(3)^ into the use of digital technologies from information systems in health activities and related areas, including telehealth, described as the provision of healthcare through ICTs to exchange information for diagnosis, treatment, research, assessment, and continuing and permanent education of professionals^(4)^.

Telehealth, previously viewed with suspicion by professionals, managers and patients, has intensified in the health sector with the coronavirus pandemic, accelerating the processes of change, such as dissemination of teleconsultations, which has led to the need for agility in the organization of services to offer this modality as well as for professional bodies to regulate it^(3)^. Globally, there has been a 50% growth in telehealth and, in Brazil, around 80%^(3)^, despite the barriers that arise for this practice, such as low financial investment, difficulties related to digital literacy, incipient continuing and permanent education actions in services, limited connectivity and interoperability, which hinder the use of health information systems, and lack of mastery by management, professionals and users in the use of digital resources and tools^(5)^.

Assuming the broad scope of the term “telehealth”, telenursing encompasses nursing consultation, interconsultation, consultancy, monitoring, health education and reception of spontaneous demand, mediated by ICTs^(6)^. Telenursing is an excellent strategy, enabling, remotely, the provision of activities and expansion of coordination and continuity of care, contributing to the qualification of nursing care, results and health outcomes for the population, and the reduction of geographical barriers to access^(2,7)^.

Considering the exponential growth of telenursing as a possibility to facilitate access and improve care planning and the identification of a gap in literature on nurses’ experience in digital health in the context of supplementary health, this article aimed to contribute to knowledge in this area, as it presents the successful experience of a service that offers care in the telehealth modality, with a view to bringing reflections and contributions to training, professional performance and healthcare. Thus, it becomes essential to know the actions and strategies used by professionals for comprehensive, resolutive and humanized care using ICTs.

## OBJECTIVES

To report the experience of telehealth in monitoring healthcare in the context of supplementary health.

## METHODS

### Ethical aspects

This study did not involve the identification of the health organization’s sector, its workers and patients who use the services provided and reported here. Formal ethical approval was waived, in accordance with Resolution 510 of April 7, 2016 of the Brazilian National Health Council.

### Study design

This is an experience report that seeks to contribute to the discussion and strengthening of nursing practice in the use of digital technologies, exercised in knowledge and experiences in the field of supplementary health. This study design is relevant to professional practice, since it provides subsidies and inspirations for applicability both for praxis and for new research.

### Study setting

The study was conducted in a specific sector of a non-profit health organization that provides supplementary healthcare in the city of São Paulo. Its staff includes six healthcare assistants (with secondary education), 13 nurses, and one family physician, who work together to ensure the coordination and continuity of healthcare, outlining individualized and person-centered plans using ICTs under the supervision of a nurse.

In the sector under analysis, nursing plays a relevant role in the care and administration dimensions of health work, managing administrative processes, people and healthcare, supported by care protocols and lines of care, technical regulations, institutional flows, information systems and team meetings, granting autonomy in decision-making in care and administrative practices.

It operates from Monday to Friday, from 7:00 a.m. to 6:00 p.m., offering only services mediated by ICTs, such as nursing and medical consultations, interconsultations, health monitoring, educational guidance, clarification of doubts and institutional flows, and reception of spontaneous demand. They occur individually or in groups, synchronously and asynchronously, and may also result in consultations shared with other professionals. Therefore, in-person care is not provided to patients. The duration of care varies, according to professional category and type of care. Care assistants have a range of five to ten minutes, and nurses and physicians, 15 to 20 minutes, carried out subsequently throughout the workday.

The profile of the population assisted encompasses all life cycles, with a greater representation of adults aged 20 to 59. Patients’ clinical conditions present different degrees of complexity in which chronic non-communicable diseases, characterized mainly by cardiovascular diseases, chronic respiratory diseases and diabetes mellitus, have a higher prevalence.

### Data collection and organization

The data were organized descriptively and chronologically based on the authors’ experience working at the institution in supervisory and coordination positions for the period between 2022 and 2024. Meeting minutes and management reports from the area were also used as sources of information. Data analysis and discussion were supported by scientific literature telehealth, jointly validated by the authors of this study.

## RESULTS

The sector in question, based on its experience with telemonitoring of patients with COVID-19 and respiratory symptoms due to the pandemic in 2020, realized that it was a powerful field for nurses to work in care mediated by digital technologies. From this previous experience, it was possible to identify some difficulties, such as the lack of integrated systems, chatbot-type messaging resources and automated reports, an insufficient number of professionals, and long response times for professionals to messages sent by patients.

In response to these challenges, and with management support for the acquisition of new technological resources, changes and expansion of the scope of operations, the sector began to offer telehealth services to patients being monitored by other healthcare teams from specialized services and Primary Healthcare in the supplementary healthcare sector, who were contracted by the institution through individual, family and corporate plans, which required an increase in the number of employees. As a result, ten nurses and one family physician were hired, while six care assistants and three nurses already in place remained, totaling 20 people on the team.

Team organization was divided into three areas based on professional category: direct reception; indirect reception; and active reception. In direct reception, the customer service assistant receives phone calls, emails or messages sent by patients from their cell phones via the WhatsApp^®^ application. When faced with questions about the flow of the institution’s services or other bureaucratic or administrative issues, professionals themselves provide the appropriate guidance or directs patients to the responsible department and concludes the service.

The indirect reception axis occurs when the demand brought by patients involve specific health issues. The care assistant then directs patients via the system to nurses, who can contact medical professionals for an interconsultation, when necessary. It is worth mentioning that patients and their families have a reference nurse; therefore, this professional has a life portfolio, as it is commonly called in supplementary health. Active care occurs when the team makes contact via message or phone call directly to patients.

Telemonitoring, the activity most frequently performed by nurses in the active sector, seeks to manage the care of patients in their different life cycles and needs, population tracking, care gap management and health risk classification through digital navigation. Other telenursing activities include receiving spontaneous demand, nursing consultations, interconsultations with other professionals and health education activities. Telenursing therefore enables the expansion of the scope of nurses’ practice in supplementary health in a scenario in which there is greater appreciation and recognition of medical practice. It also allows patients to (re)discover the role of nurses in addition to performing techniques and procedures that are more common in this sector, demonstrating the important role of these professionals in health systems.

Among the technological resources used by nurses, the following stand out: the use of electronic medical records with structured fields and electronic signature, an online task system and communication between patients-professionals and professionals-professionals, and a chatbot that, based on the answers given, directs patients to human care with a professional from the team or not.

The construction of the electronic medical record considered the acronym SOAP (S - Subjective, O - Objective, A - Assessment, P - Plan), a method for clinical records, for its structuring, based on parameterized fields. In the assessment field, classifications according to the professional category were considered. For nurses’ private use, diagnoses according to the International Classification for Nursing Practice were considered, with the possibility of using the “search” resource to select diagnosis to be considered in the care record, seeking to ensure that the nursing process stages were executed.

Taking into account the diagnoses raised, and using artificial intelligence, the system indicates which care protocol patients can be included in, such as diabetes mellitus, hypertension, obesity, prenatal care, childcare (up to 2 years of age), child and adolescent health, women’s health, transgender health, men’s health, mental health and other chronic diseases. Each protocol includes a series of pre-scheduled tasks to be performed by nurses in their “active” contacts, in order to ensure coordination and continuity of care. These tasks are parameterized in the system, generating a reminder and a list of patients to be contacted on that day by nurses so that nurses can approach them by phone, message or chatbot. It is worth mentioning that pre-scheduled tasks are guiding and aim to ensure that patients are approached between a consultation and their return visit, but they are not intended to restrict this approach. It is up to nurses, based on their clinical, critical and evidence-based reasoning, to direct and conduct care in the best way and with autonomy.

The electronic medical record with structured fields and the use of artificial intelligence enabled the construction of dashboards, reports for use by management and the care team, and process indicators (e.g., percentage of people being monitored, included in protocols, with tasks up to date and monitored by telenursing), results (e.g., percentage of patients hospitalized for causes sensitive to Primary Care and emergency room visits) and impact (e.g., mortality rate by diagnosis and clinical indicators, compared to the previous year) to ensure better care management, which are of special commercial value in supplementary health and are largely treated as confidential information by institutions.

In this context, it is worth mentioning that supplementary health has operational, financial and regulatory characteristics that are distinct from public services. It has financial resources to hire specialized advisory and consulting services, acquire software and hardware, and expand the staff and physical structure. Another particularity lies in the institutions that already have sectors in their structure such as quality and safety, technology and information, and analytics, which end up supporting the organization of new work processes.

In view of this, qualified electronic signature licenses were acquired for all nurses. The electronic medical record and online task system used in the sector were built in partnership with the institution’s outsourced technology and IT team, with nurses included in the team for development and implementation, given the need for products that better met the demands of teams in their daily practice of using health information systems. To this end, frequent meetings were held between the development teams and the sector with the involvement of management, care and administrative staff.

With the expansion of the sector’s scope of work, operational flows and care protocols were designed for care, especially telenursing, considering the existence of care protocols only for physicians and for nurses focused on the execution of techniques and procedures, a common issue in supplementary health. In this context, the care protocol stands out, with adapted guidelines and focused on telenursing practice in line with nursing diagnoses, tasks programmed in systems, complaints and most common demands in work practice, other institutional documents and Ministry of Health documents, and health guidelines, shared with the team through training and qualifications carried out on a weekly basis.

With the introduction of telehealth, there were improvements in the care provided and an increase in demand for telenursing services. Other benefits of using digital technologies include easier communication between professionals and patients, increased job satisfaction among professionals and positive assessments by patients when their health needs are met, better care management, and an understanding of the role of nurses as a leader and case manager in supplementary health, especially those with high complexity. It is also worth noting that the inclusion of nurses in frequent discussions with the technology and IT team enhanced the planning of activities that began to be carried out by the sector.

Among the difficulties encountered in the reported experience, the following stand out: connection problems; lack of skills to use different tools and systems and training focused on digital health; lack of understanding of the role of nurses by patients, other services and professionals; lack of understanding by the IT team about care and administrative team requests; and peculiarities of care in supplementary health, which is affected by the economic profile of patients, healthcare guidelines, the weak coordination of care carried out by different specialist professionals who follow a single patient and the limited articulation in the network.

## DISCUSSION

This report enables the sharing of the experience of telehealth services in supplementary health, representing a relevant and strategic cut, based on the combination of digital innovation and strategic transformation, and the important role of digital platforms as tools that enhance the assistance and management of care provided by nurses.

Nurses have been considered essential for the expansion and consolidation of new practices, such as telenursing, as they have skills for management and assistance, and knowledge of health systems and services available to guarantee coordination and continuity of care^(7)^. Furthermore, activities carried out by nurses mediated by ICTs, such as case management, definition of flows, elaboration of protocols, definition of flows, use of electronic medical records, nursing consultations, continuing health education actions, and use of health indicators, are actions and strategies that contribute to the effectiveness of care management^(2,7)^, as described in the findings of this report.

The biomedical model, which is hegemonic in the practice of different services, including supplementary health, weakens the relationship established between professional and patient, in which the latter feels without sufficient guidance after consultations. Telenursing has been presented to answer questions, provide new guidance and ensure continuity of care at different levels of health^(7)^. Nurses, when performing telenursing, facilitate safe care transitions and meet patients’ health needs, allowing for a better experience for them, promoting autonomy and better health outcomes^(2,8,9)^.

In this regard, there is a positive association between the quality of care provided and the use of digital technologies in telenursing services, constituting a differential by allowing clinical data analysis, automated decision-making, risk stratification, personalization of care and efficiency in workflows, in addition to collaborating in the reduction of diagnostic errors, waiting lists, unnecessary care in emergency and hospital services, and costs to the health system^(2,7,9)^. However, the use of software in healthcare, especially in supplementary healthcare, cannot be done solely seeking digital transformations and profit, but rather changes must be sought in people’s care experiences, both from patients’ and professionals’ perspective, supported by legislation that regulates professional practice mediated by ICTs.

As mentioned, supplementary healthcare has particularities that enabled the implementation of the experience with potential for replication in other similar services. At the same time, it presented challenges aimed at the inclusion of nurses in activities that are more common to medical professionals in this sector, such as conducting consultations, unlike what occurs in Primary Care in the public sector, especially in Family Health Strategy, in which nurses have a leadership and care management role, and are recognized by teams, users, families and the community.

Other challenges arise and are similar between public and private services, such as: interoperability between information systems; organizational culture of healthcare services; lack of skills for the digital transformation process; democratic access to technological resources and the internet; governance of health information; personalization of care with the use of mobile and wearable health devices^(5,8)^; health information life cycle management, from its generation or capture to its completion^(8)^; constant and rapid technological innovations that generate the need for professionals to learn and relearn; and lack of digital health literacy among professionals and patients for the use of different digital platforms^(10)^.

If, on the one hand, supplementary health has presented different mechanisms and strategies for creating and implementing telehealth services, on the other, the Ministry of Health has implemented others, such as initiatives promoted by the University Telemedicine Network and the Brazilian Unified System Open University, which provide training for health workers^(2)^, by the Brazilian National Health Data Network, the SUS’s main interoperability network, and by the SUS Digital Transformation Plan, which has allowed important achievements in public services, with repercussions for the population healthcare.

### Study limitations

The limitations of this study occur due to the fact that it is a local initiative that had a high financial investment for the acquisition and construction of digital technologies incorporated by the sector, which ends up portraying a specific cut, limited to the particularity of the reported experience.

### Contributions to nursing

It is expected that the results presented can support and strengthen the replication of the experience in other telehealth services, bringing implications for the work process in health and nursing, in addition to contributing to the discussion on the use of ICTs as a tool for qualifying care and digital health as a promising field of action for nurses, both in care and in the development of health software.

## FINAL CONSIDERATIONS

The study allowed sharing the experience of a telehealth service in the supplementary sector, highlighting the role of nurses in both planning and organizing a new service and developing digital products as well as in providing direct and indirect care mediated by ICTs. It reinforces the understanding that the use of different technological densities needs to be done in a way that complements each other, with adequate measurement by professionals, and that soft technologies are not disregarded, in order to guarantee comprehensive and humanized care.

Studies with other methodological designs are recommended to assess the impact of telehealth use in the care of people with different health needs, the potential and challenges in using this type of care for coordination and continuity of care, especially from patients’ perspective. It is also necessary to assess telenursing records to ensure the implementation of the nursing process and the development of specific criteria for sizing nursing staff in the provision of care mediated by digital technologies.

## Data Availability

The research data are available within the article.
