# Supplementary material for: Telehealth experience in supplementary health: monitoring health care
Source: Rev Bras Enferm. 2025 Dec 8;78(5):e20250032. doi: 10.1590/0034-7167-2025-0032 (PMC12707533; doi:10.1590/0034-7167-2025-0032)
Supplement: Translation

# Translation: Experiência de telessaúde na saúde suplementar: acompanhamento dos cuidados de saúde

**DOI:** 10.1590/0034-7167-2025-0032pt

**Authors:** Lucas Cardoso dos Santos, Glaucia Cristina de Oliveira, Natália Tatiani Gonçalves Brito, Lislaine Aparecida Fracolli

**Affiliations:** I Universidade de São Paulo. São Paulo, São Paulo, Brasil; II Sociedade Beneficente Israelita Brasileira Albert Einstein. São Paulo, São Paulo, Brasil

**Keywords:** Telessaúde, Saúde Suplementar, Acompanhamento dos Cuidados de Saúde, Sistemas de Registros Eletrônicos de Saúde, Tecnologia de Informação e Comunicação.

## Abstract

**Objetivos:**

relatar a experiência de telessaúde no acompanhamento dos cuidados em saúde no contexto da saúde suplementar.

**Métodos:**

relato de experiência desenvolvido em serviço de telessaúde na saúde suplementar referente aos anos de 2022 a 2024.

**Resultados:**

as Tecnologias de Informação e Comunicação ampliaram a oferta de cuidados, o acesso à saúde por meio da telessaúde, a busca pelos pacientes por atendimentos realizados pelo enfermeiro e a satisfação no trabalho pelos profissionais. O uso de protocolos assistenciais e tecnologias digitais possibilitou a construção de indicadores e *dashboards* para gestão do cuidado. Desafios encontrados foram problemas de conexão, falta de habilidade com as tecnologias digitais, falta de formação e literacia em saúde digital, e não compreensão do papel do enfermeiro pelo paciente, outros serviços e profissionais.

**Considerações Finais:**

a telessaúde contribui na qualificação do cuidado à saúde, evidenciando a saúde digital como campo potente de atuação para o enfermeiro.

## INTRODUÇÃO

O uso de tecnologias digitais teve seus primeiros passos durante a Primeira Guerra Mundial, com o uso do rádio para intermediar a comunicação entre médicos na frente de batalha e os hospitais. Seguiram avanços na década de 1960, com o sistema de comunicação por rádio para monitorar os sinais vitais de astronautas, nos anos 1970, com a utilização de padrões *online* para transações bancárias, seguindo para os processos industriais em 1980 e posteriormente para a área da saúde^(1)^.

No Brasil, as Tecnologias de Informação e Comunicação (TICs) são utilizadas desde 1970 com a implantação do Sistema de Informação sobre Mortalidade. Após a criação do Departamento de Informática do Sistema Único de Saúde (SUS), em 1991, outros sistemas foram implementados, somando-se a outros marcos mais recentes para a saúde digital como a promulgação da Política Nacional de Inovação Tecnológica em Saúde (2017), da Lei Geral de Proteção de Dados Pessoais (2018), da Rede Nacional de Saúde Digital (2020), da Estratégia de Saúde Digital para o Brasil (2020), da Política Nacional de Informação e Informática (2021) e da Secretaria de Informação e Saúde Digital (2023)^(2)^.

A saúde digital incorpora os avanços da tecnologia, a internet das coisas, a inteligência artificial e o *Big Data* ^(3)^ ao uso de tecnologias digitais provenientes de sistemas de informação nas atividades de saúde e áreas correlatas, incluindo a telessaúde, descrita como a prestação da assistência à saúde por meio das TICs para troca de informações para diagnóstico, tratamento, pesquisa, avaliação e educação continuada e permanente dos profissionais^(4)^.

A telessaúde, antes vista com desconfiança por profissionais, gestores e pacientes, intensificou-se na área da saúde com a pandemia do coronavírus, acelerando os processos de mudanças, como a disseminação dos teleatendimentos, o que levou à necessidade de agilidade na organização dos serviços para a oferta dessa modalidade, bem como dos órgãos de classe para sua regulamentação^(3)^. Em nível mundial, ocorreu um crescimento de 50% em telessaúde e, no Brasil, cerca de 80%^(3)^, apesar das barreiras que se apresentam para essa prática, como o baixo investimento financeiro, dificuldades correlacionadas à literacia digital, ações de educação continuada e permanente incipientes nos serviços, conectividade e interoperabilidade limitantes, que dificultam o uso dos sistemas de informação em saúde, e falta de domínio pela gestão, profissionais e usuários no uso dos recursos e ferramentais digitais^(5)^.

Assumindo a amplitude do termo “telessaúde”, a telenfermagem engloba a consulta de enfermagem, interconsulta, consultoria, monitoramento, educação em saúde e acolhimento da demanda espontânea, mediadas pelas TICs^(6)^. A telenfermagem se configura como uma excelente estratégia, possibilitando, de forma remota, a oferta de atividades e ampliação da coordenação e continuidade do cuidado, contribuindo na qualificação do cuidado em enfermagem, nos resultados e desfechos em saúde da população, e na diminuição de barreiras geográficas de acesso^(2,7)^.

Considerando o crescimento exponencial da telenfermagem como possibilidade para facilitar o acesso e melhorar o planejamento do cuidado e da identificação de uma lacuna na literatura sobre a experiência de enfermeiros na saúde digital no âmbito da saúde suplementar, este artigo visa colaborar com o conhecimento nessa área, pois apresenta a experiência exitosa de um serviço que oferta atendimentos na modalidade de telessaúde, com vistas a trazer reflexões e contribuições para a formação, atuação profissional e cuidado à saúde. Dessa forma, torna-se essencial conhecer as ações e estratégias utilizadas pelos profissionais para uma assistência integral, resolutiva e humanizada com uso das TICs.

## OBJETIVOS

Relatar a experiência de telessaúde no acompanhamento dos cuidados em saúde no contexto da saúde suplementar.

## MÉTODOS

### Aspectos éticos

O presente estudo não envolveu a identificação do setor da organização de saúde, de seus trabalhadores e de pacientes que utilizam os serviços prestados e aqui relatados. Dispensou-se a aprovação ética formal, conforme Resolução nº 510, de 07 de abril de 2016, do Conselho Nacional de Saúde.

### Tipo de estudo

Trata-se de relato da experiência que busca contribuir para a discussão e fortalecimento da prática do enfermeiro no uso das tecnologias digitais, exercitada nos saberes e experiências no âmbito da saúde suplementar. Este tipo de estudo tem relevância para a prática profissional, uma vez que traz subsídios e inspirações de aplicabilidade tanto para a *práxis* quanto para novas pesquisas.

### Cenário do estudo

O local deste estudo é um setor específico de uma organização de saúde sem fins lucrativos, que atua na saúde suplementar, no município de São Paulo. Tem em sua composição seis assistentes de atendimento (nível médio de formação), 13 enfermeiros e um médico de família, que atuam em conjunto para garantir a coordenação e continuidade do cuidado à saúde, delineando planos individualizados e centrados na pessoa com a utilização de TICs sob a supervisão de uma enfermeira.

No setor em análise, a enfermagem tem papel relevante nas dimensões assistir e administrar do trabalho em saúde, realizando a gestão de processos administrativos, de pessoas e do cuidado à saúde, apoiada em protocolos assistenciais e linhas de cuidados, regulamentos técnicos, fluxos institucionais, sistemas de informação e reuniões de equipe, conferindo autonomia na tomada de decisão nas práticas assistenciais e administrativas.

O seu funcionamento ocorre de segunda a sexta-feira, das 07:00 às 18:00, ofertando apenas atendimentos mediados pelas TICs, como consultas de enfermagem e médica, interconsultas, monitoramento em saúde, orientações educativas, esclarecimento de dúvidas e dos fluxos institucionais, e acolhimento da demanda espontânea. Ocorrem de forma individual ou em grupo, síncrona e assíncrona, podendo ainda resultar em consultas compartilhadas com outros profissionais. Portanto, não são realizados atendimentos presenciais aos pacientes. O tempo de duração dos atendimentos é variável, conforme categoria profissional e tipo de atendimento. Os assistentes de atendimentos apresentam variação de cinco a dez minutos, e enfermeiros e médico, de 15 a 20 minutos, realizados de forma subsequente ao longo da jornada de trabalho.

O perfil da população atendida engloba todos os ciclos de vida, com maior representação de adultos com 20 a 59 anos. As condições clínicas dos pacientes apresentam diferentes graus de complexidade em que as doenças crônicas não transmissíveis, caracterizadas principalmente pelas doenças cardiovasculares, doenças respiratórias crônicas e diabetes mellitus, apresentam maior prevalência.

### Coleta e organização dos dados

Os dados foram organizados, de forma descritiva e cronológica, a partir da experiência dos autores com atuação na instituição, em cargos de supervisão e coordenação, referente ao período compreendido entre 2022 e 2024. Utilizaram-se também como fonte de informações as atas das reuniões e os relatórios gerenciais da área. A análise e discussão dos dados foram apoiadas na literatura científica que aborda a temática da telessaúde com validação conjunta da autoria deste estudo.

## RESULTADOS

O setor em questão, a partir da experiência com o telemonitoramento de pacientes com COVID-19 e sintomáticos respiratórios em decorrência da pandemia em 2020, percebeu ser campo potente para a atuação do enfermeiro no cuidado mediado por tecnologias digitais. Dessa experiência prévia, foi possível identificar algumas dificuldades, como a falta de sistemas integrados, de recursos de mensagens do tipo *chatbot* e de relatórios automatizados, número insuficiente de profissionais, e tempo longo de resposta dos profissionais às mensagens enviadas pelos pacientes.

A partir desses desafios, e com o apoio da gestão para aquisição de novos recursos tecnológicos, mudanças e ampliação no escopo de atuação, o setor passou a ofertar atendimentos na modalidade de telessaúde aos pacientes em acompanhamento com outras equipes de saúde de serviços especializados e da Atenção Primária à Saúde do setor da saúde suplementar, conveniados à instituição por meio de planos individuais, familiares e empresariais, o que demandou aumento no quadro de funcionários. Dessa forma, foram contratados dez enfermeiros e um médico de família, mantendo os seis assistentes de atendimento e os três enfermeiros já existentes, totalizando 20 pessoas na equipe.

A organização da equipe foi dividida a partir da categoria profissional em três eixos: receptivo direto; receptivo indireto; e ativo. No receptivo direto, o assistente de atendimento recebe ligações telefônicas, *e-mail* ou mensagens enviadas pelos pacientes de seus celulares pelo aplicativo *WhatsApp^®^*. Quando diante de dúvidas sobre o fluxo dos serviços da instituição ou outras questões burocráticas ou administrativas, o próprio profissional realiza as orientações cabíveis ou direciona para o setor responsável e finaliza o atendimento.

O eixo do receptivo indireto se dá quando a demanda trazida pelo paciente envolve questões específicas de saúde. O assistente de atendimento então direciona via sistema ao enfermeiro, que pode acionar o profissional médico para uma interconsulta, quando necessário. Vale mencionar que os pacientes e suas famílias possuem um enfermeiro de referência; portanto, esse profissional tem uma carteira de vidas, como é chamado comumente na saúde suplementar. Já os atendimentos ativos ocorrem quando a equipe realiza o contato por mensagem ou ligação diretamente ao paciente.

O telemonitoramento, atividade mais desenvolvida pelos enfermeiros do setor no eixo ativo, busca, por meio da navegação digital, a realização da gestão do cuidado dos pacientes assistidos nos seus diferentes ciclos de vida e necessidades, o rastreamento populacional, o gerenciamento de *care gaps* e a classificação de risco de saúde. Outras atividades de telenfermagem realizadas envolvem acolhimento da demanda espontânea, consulta de enfermagem, interconsulta com outros profissionais e ações de educação na saúde. A telenfermagem possibilita, então, a ampliação do escopo de prática do enfermeiro na saúde suplementar em um cenário em que há uma maior valorização e reconhecimento da prática médica. Permite, também, que o paciente (re)conheça a atuação do enfermeiro para além da realização de técnicas e procedimentos, mais comuns nesse setor, demonstrando o papel relevante desse profissional nos sistemas de saúde.

Entre os recursos tecnológicos utilizados pelo enfermeiro, destacam-se o uso de prontuário eletrônico com campos estruturados e assinatura eletrônica, sistema de tarefas *online* e de comunicação entre pacientes-profissionais e profissionais-profissionais, e *chatbot* que, a partir de respostas dadas, direciona ou não o paciente para atendimento humano com profissional da equipe.

A construção do prontuário eletrônico considerou o acrônimo SOAP (S - Subjetivo, O -Objetivo, A - Avaliação, P - Plano), método para registro clínico, para sua estruturação, a partir de campos parametrizados. No campo avaliação, foram consideradas classificações segundo a categoria profissional. Para uso privativo do enfermeiro, foram considerados os diagnósticos segundo a Classificação Internacional para a Prática de Enfermagem, com a possibilidade de utilização do recurso “pesquisar” para a seleção do diagnóstico a ser considerado no registro do atendimento, buscando garantir que as etapas do processo de enfermagem fossem executadas.

Em consideração aos diagnósticos levantados, e com uso de inteligência artificial, o sistema indica qual protocolo assistencial o paciente pode ser incluído, como diabetes mellitus, hipertensão arterial sistêmica, obesidade, pré-natal, puericultura (até 2 anos de idade), saúde da criança e adolescente, saúde da mulher, saúde da pessoa transgênero, saúde do homem, saúde mental e outras doenças crônicas. Cada protocolo contempla uma série de tarefas pré-agendadas a serem executadas pelo enfermeiro em seus contatos “ativos”, de forma a garantir a coordenação e continuidade do cuidado. Essas tarefas são parametrizadas em sistema, gerando um lembrete e uma relação de pacientes a serem contatados no dia pelo enfermeiro, para que esse possa abordá-los por ligação, mensagem ou *chatbot*. Vale ressaltar que as tarefas pré-agendadas são norteadoras e visam garantir que o paciente seja abordado entre uma consulta e seu retorno, mas não tem a pretensão de engessar essa abordagem, cabendo ao enfermeiro, a partir do seu raciocínio clínico, crítico e baseado em evidências, direcionar e conduzir o atendimento da melhor forma e com autonomia.

O prontuário eletrônico com campos estruturados e uso de inteligência artificial possibilitaram a construção de *dashboards*, relatórios para uso da gestão e da equipe assistencial e de indicadores de processo (*e.g.*, porcentagem de pessoas em acompanhamento, inseridas em protocolos, com tarefas em dia e acompanhadas por telenfermagem), de resultado (*e.g.*, porcentagem de pacientes internados por causas sensíveis à Atenção Primária e passagem no pronto-socorro) e de impacto (*e.g.*, razão de mortalidade por diagnóstico e de indicadores clínicos, com comparação ao ano anterior), para garantia de um melhor gerenciamento do cuidado, sendo esses de especial valor comercial na saúde suplementar e tratados em grande parte como informações sigilosas pelas instituições.

Nessa conjuntura, menciona-se que a saúde suplementar apresenta características operacionais, financeiras e regulatórias distintas dos serviços públicos. Apresenta recursos financeiros para contratação de serviços especializados para assessoria e consultoria, aquisição de *softwares* e *hardwares,* e ampliação do quadro de profissionais e da estrutura física. Outra particularidade reside nas instituições que já apresentam em sua estrutura setores como de qualidade e segurança, tecnologia e informação e *analytics*, que acabam por apoiar a organização de novos processos de trabalho.

À vista disso, foram adquiridas licenças de assinaturas eletrônicas qualificadas para todos os enfermeiros. O prontuário eletrônico e o sistema de tarefas *online* utilizados no setor foram construídos em parceria com a equipe terceirizada de tecnologia e informática da instituição, com a inclusão de enfermeiros na equipe para o desenvolvimento e implementação, visto a necessidade de produtos que atendessem melhor às demandas das equipes na prática diária no uso dos sistemas de informação em saúde. Para tal, reuniões frequentes foram realizadas entre as equipes de desenvolvimento e o setor com envolvimento da gestão, equipe assistencial e administrativa.

Com a expansão do escopo de trabalho do setor, foi pensada para os atendimentos, sobretudo de telenfermagem, a construção de fluxos operacionais e protocolos assistenciais, considerando a existência de protocolos assistenciais apenas para médicos e para a enfermagem voltados à execução de técnicas e procedimentos, questão comum na saúde suplementar. Nesse contexto, destacam-se o protocolo assistencial, com orientações adaptadas e voltadas para prática da telenfermagem em consonância com os diagnósticos de enfermagem, tarefas programadas em sistemas, queixas e demandas mais comuns na prática do trabalho, outros documentos institucionais e do próprio Ministério da Saúde, e *guidelines* da área da saúde, compartilhados com a equipe por meio de treinamentos e capacitações realizados com frequência semanal.

A partir da oferta da telessaúde, perceberam-se melhorias no cuidado ofertado e aumento da procura pelo teleatendimento de enfermagem. Outros benefícios no uso das tecnologias digitais residem na facilidade de comunicação entre profissionais e pacientes, no aumento da satisfação no trabalho pelos profissionais e de avaliações positivas pelos pacientes, ao terem suas necessidades de saúde atendidas, no melhor gerenciamento do cuidado, e na compreensão do papel do enfermeiro como líder e gestor de casos na saúde suplementar, sobretudo aqueles com alta complexidade. Destaca-se ainda que a inclusão do enfermeiro nas discussões frequentes com a equipe de tecnologia e informática potencializou a concretização do planejamento das atividades que passaram a ser realizadas pelo setor.

Entre as dificuldades encontradas na experiência relatada, citam-se: problemas de conexão; falta de habilidade para uso das diferentes ferramentas e sistemas e de formação voltada à saúde digital; não compreensão do papel do enfermeiro pelo paciente, outros serviços e profissionais; não compreensão da equipe de informática sobre acerca das solicitações da equipe assistencial e administrativa; e peculiaridades do atendimento na saúde suplementar, que é afetado pelo perfil econômico dos pacientes, pelas diretrizes de atenção à saúde, pela frágil coordenação do cuidado realizada por diferentes profissionais especialistas que acompanham um único paciente e pela limitada articulação em rede.

## DISCUSSÃO

O presente relato possibilita o compartilhamento da experiência de serviço de telessaúde na saúde suplementar, representando recorte relevante e estratégico, a partir da combinação de inovação digital e transformação estratégica, e o papel importante das plataformas digitais como ferramentas que potencializam a assistência e o gerenciamento do cuidado realizados pelo enfermeiro.

Enfermeiros têm sido considerados essenciais para a expansão e consolidação de novas práticas, como a telenfermagem, por possuírem competência para a gestão e assistência, e conhecimento dos sistemas e serviços de saúde disponíveis para a garantia da coordenação e continuidade do cuidado^(7)^. Além disso, atividades realizadas por enfermeiros mediadas pelas TICs, como a gestão de casos, definição de fluxos, elaboração de protocolos, definição de fluxos, utilização de prontuários eletrônicos, consultas de enfermagem, ações de educação permanente em saúde e uso de indicadores de saúde, são ações e estratégias que contribuem para efetivação da gestão do cuidado^(2,7)^, como descrito nos achados deste relato.

O modelo biomédico, hegemônico na prática dos diferentes serviços, incluindo a saúde suplementar, fragiliza a relação estabelecida entre profissional e paciente, em que esse último se sente sem orientação suficiente após os atendimentos. A telenfermagem tem se apresentado para acolher dúvidas, realizar novas orientações e garantir a continuidade do cuidado em diferentes níveis de saúde^(7)^. Enfermeiros, ao realizarem a telenfermagem, viabilizam transições de cuidado seguras e atendem às necessidades de saúde pelos pacientes, permitindo uma melhor experiência para os mesmos, com promoção da autonomia e melhores resultados em saúde^(2,8,9)^.

Nesse sentido, existe uma associação positiva entre a qualidade da assistência prestada e o uso de tecnologias digitais nos serviços de telenfermagem, constituindo um diferencial ao permitir a análise de dados clínicos, tomada de decisão automatizada, estratificação de risco, personalização do atendimento e eficiência nos fluxos de trabalho, além de colaborar na redução de erros diagnósticos, listas de espera, atendimentos desnecessários em serviços de emergência e hospitalares, e custos ao sistema de saúde^(2,7,9)^. Todavia, o uso de *softwares* na saúde, especialmente na saúde suplementar, não pode ser feito buscando unicamente transformações digitais e lucro, mas devem-se buscar mudanças nas experiências de cuidado das pessoas, tanto na perspectiva do paciente quanto do profissional, apoiadas nas legislações que regulamentam a prática profissional mediada pelas TICs.

Como mencionado, a saúde suplementar apresenta particularidades que possibilitaram a implementação da experiência com potencial de replicação para outros serviços similares. Ao mesmo tempo, apresentou desafios voltados à inserção do enfermeiro em atividades mais comuns ao profissional médico nesse setor, como a realização de consultas, diferente do que ocorre na Atenção Primária no setor público, sobretudo na Estratégia Saúde da Família, em que o enfermeiro possui papel de liderança e de gestor do cuidado, e reconhecimento pelas equipes, usuários, famílias e comunidade.

Outros desafios se apresentam e são similares entre serviços públicos e privados como: a interoperabilidade entre os sistemas de informação; a cultura organizacional dos serviços da saúde; a falta de competências para o processo de transformação digital; o acesso democrático aos recursos tecnológicos e à internet; a governança da informação em saúde; a personalização do cuidado com a utilização de dispositivos móveis e vestíveis no âmbito da saúde^(5,8)^; a gestão do ciclo de vida da informação em saúde, envolvendo desde a sua geração ou captura até a sua terminação^(8)^; as constantes e rápidas inovações tecnológicas que geram a necessidade dos profissionais aprenderem e reaprenderem; e a falta de literacia em saúde digital pelos profissionais e pacientes para uso das diferentes plataformas digitais^(10)^.

Se, por um lado, a saúde suplementar tem apresentado diferentes mecanismos e estratégias para criação e implantação de serviços de telessaúde, por outro, o Ministério da Saúde tem implementado outras, como as iniciativas promovidas pela Rede Universitária de Telemedicina e pela Universidade Aberta do Sistema Único do Brasil, que oportunizam a formação de trabalhadores na área da saúde^(2)^, pela Rede Nacional de Dados em Saúde, principal rede de interoperabilidade do SUS, e pelo Plano de Transformação Digital do SUS, que tem permitido conquistas importantes nos serviços públicos, com repercussão para o cuidado à saúde da população.

### Limitações do estudo

As limitações deste estudo ocorrem pelo fato de ser uma iniciativa local que teve alto investimento financeiro para aquisição e construção de tecnologias digitais incorporadas pelo setor, o que acaba por retratar um recorte específico, limitado à particularidade da experiência relatada.

### Contribuições para a área da enfermagem

Espera-se que os resultados apresentados possam subsidiar e fortalecer a replicação da experiência em outros serviços de telessaúde, trazendo implicações para o processo de trabalho em saúde e enfermagem, além de contribuir para a discussão sobre o uso das TICs como ferramenta de qualificação do cuidado e da saúde digital como um campo promissor de atuação para o enfermeiro, tanto na assistência quanto no desenvolvimento de *softwares* em saúde.

## CONSIDERAÇÕES FINAIS

O estudo permitiu compartilhar a experiência de um serviço de telessaúde do setor suplementar, com destaque para a atuação do enfermeiro tanto no planejamento e organização de um novo serviço e desenvolvimento de produtos digitais quanto na prestação de cuidados diretos e indiretos mediados pelas TICs. Reforça-se a compreensão de que o uso de diferentes densidades tecnológicas precisa ser feito de forma que uma complemente a outra, com adequada medida pelos profissionais, e que as tecnologias leves não sejam desconsideradas, de modo a garantir um cuidado integral e humanizado.

Recomendam-se estudos com outros desenhos metodológicos que permitam avaliar o impacto do uso da telessaúde no cuidado às pessoas com suas diferentes necessidades de saúde, as potencialidades e os desafios no uso dessa modalidade de atendimento para coordenação e continuidade do cuidado, sobretudo na perspectiva dos pacientes. Faz-se necessário, também, a avaliação dos registros de telenfermagem, de forma a garantir a implementação do processo de enfermagem e a elaboração de critérios específicos para o dimensionamento de pessoal de enfermagem na oferta de cuidados mediados por tecnologias digitais.

## Data Availability

Os dados da pesquisa estão disponíveis no corpo do artigo.
